# LGBTQI content on obstetrics and gynecology residency websites

**DOI:** 10.1186/s12909-023-04624-3

**Published:** 2023-11-12

**Authors:** Amythis Soltani, Saman Baban, Katherine French, Kathryn Annand, Annemarie Pelle, Bianca M. Stifani

**Affiliations:** Department of Obstetrics & Gynecology, New York Medical College / Westchester Medical Center, Valhalla, NY USA

**Keywords:** Residency, Medical education, Obstetrics, Gynecology, LGBTQI, Lesbian, Gay, Transgender, Intersex

## Abstract

**Background:**

In the United States (US), many obstetrics & gynecology (OB-GYN) trainees feel unprepared to care for lesbian, gay, bisexual, transgender, queer, and intersex (LGBTQI) individuals, but interest in this topic is rising. Residency program websites are one way that directors can advertise whether this training is offered within their program. We aimed to describe the extent to which LGBTQI content is currently featured on OB-GYN residency websites across the country.

**Methods:**

We identified all OB-GYN residency programs in the United States using a publicly available database. We systematically searched for select LGBTQI keywords on program websites. We collected data on mentions of LGBTQI didactics and rotations. We also searched whether LGTBQI keywords were included in diversity, equity, and inclusion (DEI) statements. We used multivariable logistic regression to compare the characteristics of programs that do and do not include this content. We used STATA SE Version 16.0 for all analyses and set the level of significance at 5%.

**Results:**

We included 287/295 US OB-GYN residency programs in our analysis (97.3%) and excluded 8 that did not have websites. We identified any LGBTQI content on 50 program websites (17.4%), and specific mention of didactics or rotations on 8 websites (2.8%). On multivariable analysis, programs in the West were more likely to include any LGBTQI content compared to programs in the South (OR 2.81, 95%CI 1.04–7.63), as were programs with 1 or 2 fellowships (OR 3.41, 95%CI 1.43–8.14) or 3 or more fellowships (OR 4.85, 95%CI 2.03–11.57) compared to those without fellowships. Programs in departments led by female chairs were also more likely to include LBTQI content (OR 3.18, 95%CI 1.55–6.51).

**Conclusions:**

Academic programs, West Coast programs, and those with departments led by female chairs are more likely to mention LGBTQI keywords on their websites. Given the increasing interest in LGBTQI education for OB-GYN trainees, program directors should consider providing training opportunities and including this content on their websites.

**Supplementary Information:**

The online version contains supplementary material available at 10.1186/s12909-023-04624-3.

## Background

Lesbian, gay, bisexual, transgender, queer, and intersex (LGBTQI) individuals account for approximately 7.1% of the population in the United States [1]. Research demonstrates that members of this population experience poorer mental and physical health outcomes [2]. Many factors contribute to healthcare disparities in the LGBTQI population. These include social norms prioritizing heterosexuality, discrimination at the individual and institutional level, and stigma among healthcare providers and in communities at large [3]. Issues of health equity and access to care for LGBTQI individuals are relevant to all medical specialties, but the field of obstetrics and gynecology holds a particularly challenging position because it traditionally caters only to female patients [3].

One recent study found that less than half of board-certified OB-GYNs reported having received any training in LGBTQI health during residency [4]. Other survey studies have found that many OB-GYN residents feel unprepared to care for lesbian, bisexual, and transgender patients [5, 6]. However, residents desire more education on how to deliver care to LGBTQI patients [7]. Given OB-GYNs typically address sexual health within their practice, it is vital that future generations of OB-GYNs are adequately trained to care for all patients.

This increasing interest in expanding LGBTQI-related education and training in OB-GYN residency is a promising first step in preparing OB-GYNs to care for this patient population. Medical students who are applying to residency and desire training in LGBTQI health-related issues can use program websites to learn whether programs offer training in this area. Residency websites are publicly accessible and may reflect training priorities and institutional values. During the COVID-19 pandemic, the residency interview process radically changed, as virtual interviews replaced travel to in-person interviews. In this context, online informational material about programs is likely to play an even more important role in the residency selection process.

We undertook a review of United Stated-based OB-GYN residency program websites to determine the proportion of programs that include a mention of training in LGBTQI health on their website. We also compared the characteristics of residency programs that do and do not explicitly mention training in LGBTQI health.

## Method

This is a cross-sectional analysis of all OB-GYN residency program websites in the United States. We systematically examined each residency program website from November 1st to November 22nd, 2021, searching for specific LGBTQI keywords. We excluded programs with no existing website from the analysis.

### Data collection

We identified all OB-GYN residency programs in the United States using the American Medical Association’s Residency & Fellowship Programs Database (FREIDA). Two researchers then examined each program website separately to search for LGBTQI content. A third reviewer resolved any conflicts or discrepancies between reviewers. We searched the website home page and all additional sub-pages using the following LGBTQI-specific keywords: LGBT/LGTBQ/LGBTQI, transgender, trans health, queer, lesbian/gay/bisexual. We selected similar keywords to those used in existing research assessing LGBTQI healthcare content online [8].

Using our keyword searches, we determined whether programs websites had any mention of LGBTQI health in any portion of the website; mention of LGBTQI-specific rotations or didactics, mention of LGBTQI health in the program’s diversity, equity, and inclusion (DEI) statement, or other mentions of LGBTQI health. We also collected quotes from the websites that reflected this content.

We collected various program characteristics such as location (city, state, region) and program type (community, university based, etc.) from FRIEDA [9]. We also looked at the extent of program websites, and defined them as simple, moderate, or complex. We defined a simple website as having no subpages, a moderate website as having less than or equal to 5 subpages, and a complex website as having greater than 5 subpages. We used the program website to determine number of residents, number of fellowships available, sex of department chair, and sex of program director. Of note, we based the sex of the chair and program director on external characteristics and/or the individual’s name as we were unable to collect data on the individuals’ gender identities. To determine state political party, we looked at the last five presidential elections and assigned party based on election results. We labeled states in which no one party won more than three of the last five presidential elections as swing states.

### Data analysis

We created a dichotomous variable for any mention of LGBTQI health (whether didactics, rotations, or mention in DEI statement, or “other” mention of LGBTQI content) versus none (primary outcome). An example of the “other” category is mention of LGBTQI research. We also created a dichotomous secondary outcome that only included mention of LGBTQI health didactics or rotations, to ensure that we had specific information about training.

We conducted descriptive statistical analyses of variables reflecting program characteristics. To compare the characteristics of programs that did and did not include LGBTQI content, we initially used the Chi Square or Fisher’s Exact tests. To examine the predictors of having any LGBTQI content on the program website (primary outcome), and of having LGBTQI training (rotation or didactics, secondary outcome), we constructed two multivariable logistic regression models. To do so, we initially included all variables with p < 0.1 on univariable analyses. We then build multivariable regression models using a stepwise, backward selection approach. We first included all covariates with p < 0.25 on bivariate analysis and then sequentially removed covariates with the highest p value until all variables were significant.

We conducted all analyses using STATA Version 16.0 and set the significance level at 5%.

### Ethical approval

This research study does not involve human subjects. As such, the New York Medical College Institutional Review Board deemed the study exempt from review.

## Results

**Fig. 1:**
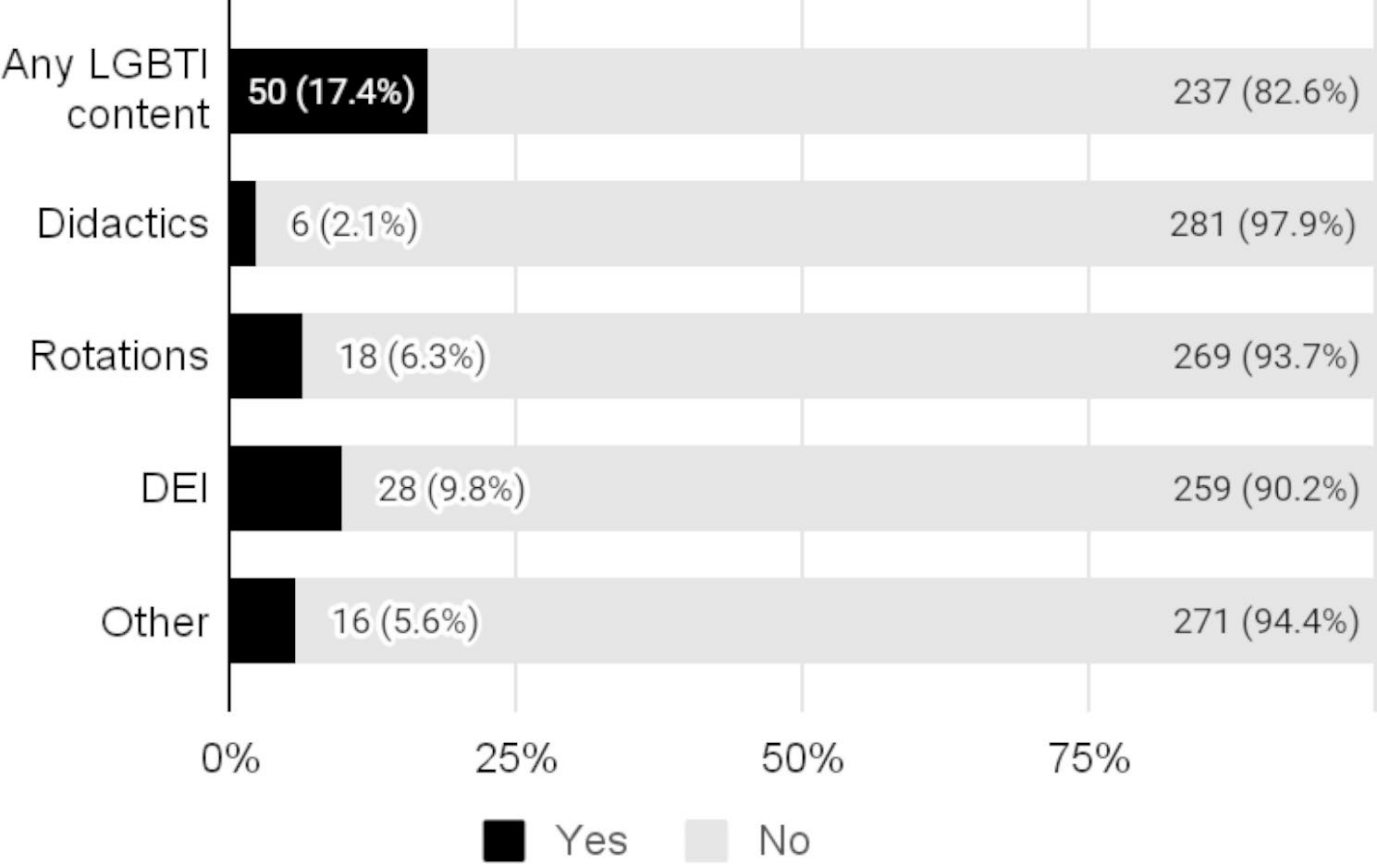
Presence of lesbian, gay, bisexual, transgender, queer, intersex (LGBTQI) content on 287 United States Obstetrics and gynecology residency program websites All reported values are n (%). DEI: diversity, equity, and inclusion statement

We identified 295 OB-GYN residency programs through the FREIDA database. Of these, we excluded eight programs that did not have a website. Only 50 (17.4%) program websites included any LGBTQI content. Only six program websites (2.1%) mentioned LGBTQI-related didactics, 18 (6.3%) mentioned LGBTQI-specific rotations, 28 (9.8%) contained LGBTQI content in their DEI statements, and 16 (5.6%) had mention of LGBTQI content in another context (Fig. 1).

Most of the 287 websites we reviewed were complex (n = 208, 72.5%). Almost half of programs were university-based (n = 124, 43.2%), while some others (n = 109, 37.9%) were community-based university-affiliated. Most programs did not have any religious affiliation declared on their websites (n = 260, 90.6%). Approximately two thirds of program directors were female (n = 182, 63.4%), while only 28.6% (n = 82) of department chairs were female. Table 1 shows the characteristics of the 287 programs included in the study.

**Table 1:** Characteristics of United States Obstetrics and gynecology residency programs included in a review of lesbian, gay, bisexual, transgender, queer, intersex content on program websites

	n (%)
**Region (n = 295)**	
Northeast	84 (28.5)
Midwest	71 (24.1)
South	100 (33.9)
West	37 (12.5)
Puerto Rico	3 (1.0)
**State Party (n = 295)**	
Republican	80 (27.4)
Democratic	130 (44.5)
Swing	85 (28.8)
**Program Type (n = 295)**	
Community	55 (18.6)
University	124 (42.0)
Community-based University-affiliated	109 (37.0)
Military	7 (2.4)
**Has Website (n = 295)**	
Yes	287 (97.3)
No	8 (2.7)
**Website Extent (n = 287)**	
Simple	24 (8.4)
Moderate	55 (19.2)
Complex	208 (72.4)
**Religious Affiliation (n = 287)**	
No	260 (90.5)
Yes	25 (8.7)
Unsure	2 (0.8)
**# of Residents (n = 287)**	
<16	64 (22.3)
16–24	155 (54.0)
>24	68 (23.7)
**# of Fellowships (n = 287)**	
0	158 (55.1)
1–2	67 (23.3)
3+	62 (21.6)
**Sex of Program Director (n = 287)**	
Male	100 (34.9)
Female	182 (63.4)
N/A	5 (1.7)
**Sex of Chair (n = 287)**	
Male	143 (49.8)
Female	82 (28.6)
N/A	62 (21.6)

On univariable analysis, region (p = 0.037), program type (p = 0.009), website extent (p = 0.009), number of residents (p = 0.019), number of fellowships (p = < 0.001), sex of program director (p = 0.023), and sex of department chair (p = < 0.001) were significantly associated with mention of LGBTQI health [Table 2]. Table 1 in the Supplementary material shows the univariable analysis for the secondary outcome (didactics and rotations only). Program region (p = 0.009), website extent (p = 0.033), number of residents (p = 0.021), number of fellowships (p < 0.001), sex of program director (p = 0.021), and sex of department chair (p = 0.004) were associated with mention of LGBTQI health didactics or rotations.

**Table 2:** Association between presence of lesbian, gay, bisexual, transgender, queer, intersex content US OB-GYN residency websites and program characteristics

	Any mention of LGBTQI content (n = 50)	No Mention of LGBTQI content (n = 237)	
	n (%)	n (%)	p value*
**Region**			0.037
Northeast	14 (17.3)	67 (82.7)	
Midwest	11 (15.3)	61 (84.7)	
South	12 (12.4)	85 (87.6)	
West	13 (35.1)	24 (64.9)	
**State Party**			0.216
Democratic	27 (21.1)	101 (78.9)	
Republican	9 (11.4)	70 (88.6)	
Swing	14 (17.5)	66 (82.5)	
**Program Type**			0.009
Community	7 (13.5)	45 (86.5)	
University	32 (25.8)	92 (74.2)	
CB University	11 (10.6)	93 (89.4)	
Military	0 (0)	7 (100)	
**Website Extent**			0.009
Simple	1 (4.2)	23 (95.8)	
Moderate	4 (7.3)	51 (92.7)	
Complex	45 (21.6)	163 (78.4)	
**# of Residents**			0.019
<16	5 (7.8)	59 (92.2)	
16–24	27 (17.4)	128 (82.6)	
>24	18 (26.5)	50 (73.5)	
**# of Fellowships**			< 0.001
0	11 (7.0)	147 (93.0)	
1 or 2	17 (25.4)	50 (74.6)	
3+	22 (35.5)	40 (64.5)	
**Sex of Program Director**			0.023
Male	10 (10.0)	90 (90.0)	
Female	40 (22.0)	142 (78.0)	
Unknown	0 (0)	5 (100)	
**Sex of Chair**			< 0.0001
Male	18 (12.6)	125 (87.4)	
Female	28 (34.2)	54 (65.9)	
Unknown	4 (6.5)	58 (93.6)	

LGBTQI: lesbian, gay, bisexual, transgender, queer, intersex. Values are n (%). Percentages are row percentages. * P-values based on Chi-square or Fisher’s Exact tests

Table 3 shows the results of a multivariable logistic regression model examining predictors of any mention of LGBTQ content. The covariates included in the final model were program region, number of fellowships, and chair sex. The odds of mentioning LGBTQI health among programs in the West compared to those in the South were 2.81 (95%CI 1.04–7.63, p = 0.042). For programs with one or two fellowships compared to no fellowships, the odds of mentioning LGBTQI health were 3.41 (95% CI 1.43–8.14, p = 0.006). Programs with 3 or more fellowships had 4.85 times the odds of mentioning LGBTQI content (95%CI 2.03–11.57, p < 0.001). For programs with a female department chair compared to those with a male chair, the odds ratio of mentioning LGBTQI health was 3.18 (95% CI 1.55–6.51, p = 0.002).

On multivariable analysis, the only significant predictor of mentioning LGTBQI didactics or rotations specifically (secondary outcome) was number of fellowships. Programs with one or two fellowships had 12.1 times the odds of including this content compared to those with no fellowships (95%CI 2.54–57.69, p = 0.002), while programs with 3 or more fellowships had 18.72 times the odds (95%CI 4.05–86.49, p < 0.001).

**Table 3:** Results of a multivariable logistic regression model examining predictors of LGBTQI content on US OB-GYN residency program websites

	OR	95%CI	p value*
**Region**			
South	*ref*	*ref*	*ref*
West	2.81	1.04–7.63	0.042
Northeast	1.23	0.50-3.00	0.657
Midwest	1.63	0.63–4.21	0.316
Puerto Rico	1		
**Number of fellowships**			
None	*ref*	*ref*	*ref*
1 or 2	3.41	1.43–8.14	0.006
3 or more	4.85	2.03–11.57	< 0.001
**Chair sex**			
Male	*ref*	*ref*	*ref*
Female	3.18	1.55–6.51	0.002
Unknown	0.76	0.23–2.52	0.649

Results based on logistic regression model including the variables shown

## Discussion

In this review of LGBTQI content on US OB-GYN residency program websites, we found that less than a fifth of OB-GYN residency websites had any content related to the health of LGBTQI communities. In order to understand how these findings may relate to education opportunities, we also looked at the mention of LGBQI specific rotations and didactics, which were even more rare. Although residency program websites do not necessarily reflect all training opportunities, they are important for applicants who are deciding where to train, as has been demonstrated in other specialties [10, 11]. Our findings are at odds with the fact that most OB-GYN residents surveyed in the past have expressed interest in learning how to care for the LGBTQI population [5].

We also found that certain program characteristics are associated with mentions of LGBTQI content on program website: program region (West Coast particularly), number of fellowships, and female sex of the department chair. We interpreted the number of fellowships as a marker of academic status. These results are new to the literature, but they are not surprising. A review of online directory for gender affirmation surgery found that a disproportionate percentage of surgeons providing these services practice in the West (30.5%) and Northeast (25.6%), despite this geographic distribution not matching the distribution of the patient population that would benefit from these services [12]. A national survey concluded that providing gender-affirming care was more common among OB-GYN practices in the West than in the Northeast, Midwest and South [13].

Our study has several limitations. First, we are unable to assess how website content correlates with actual training experiences. It is difficult to establish how many programs offer LGBTQI training because this may not always be advertised on program websites. Residency websites may also be outdated and may not reflect new training opportunities. In a 2016–2017 survey of OB-GYN residency program directors, 51% stated that their program offered transgender health education or training. However, only 61% of those invited to complete the survey did so, and it is possible that nonrespondents were less likely to offer this training [14]. If this is the case, our study demonstrates a discrepancy between training experiences and what is reflected on program websites. Additionally, this may reflect institution values and continues to highlight possible limitations in residency recruitment.

Another limitation is that we conducted our search at a specific point in time and our findings may not reflect changes that have occurred since we completed data collection. Finally, we did not assess programs’ social media platforms (such as Instagram or Facebook) for LGBTQI-related content. A study of ENT programs found that higher ranked programs are more active on social media, have more followers, and adopt social media earlier [15]. Another study of orthopedics programs found that the program’s Instagram was used by most applicants (61.9%) to learn about programs and nearly a third rely on it as their main resource when researching prospective programs [16]. In the current climate of virtual residency interviews due to the COVID-19 pandemic, social media has become an increasingly popular means for applicants to learn about residency programs across various specialties [17]. These platforms may include content that is more up-to-date and in line with current training opportunities. Another limitation is the LGBQTI keywords used may not reflect all words used by residency program websites to discuss such training opportunities and content.

Our study also has several strengths. First, our approach is novel as to our knowledge there have been no systematic assessments of US OB-GYN residency program websites. Second, we systematically reviewed all residency programs in the United States using established keywords. Lastly, we collected all the data in a short time period, and were therefore able to obtain a snapshot of all US programs at a specific point in time.

## Conclusions

In conclusion, we found that LGBTQI content exists on a small minority of US OB-GYN residency program websites. Currently, all OB-GYN residency recruitment efforts are virtual as means to improve access to residency interview opportunities for applications. Therefore, medical students applying to residency can use websites to learn whether programs offer training in LGBQTI health. Many applicants are using websites to understand and reflect on program and institutional values. The lack of LGBQTI health training mentioned on program websites as demonstrated by our study may reflect a lack of interest within the institution to highlight this training, even if it is available. In order to better serve the LGBQTI and recruit residents interested in providing healthcare to this community, programs may consider marketing their complete educational experiences and more thoroughly documenting their training experiences in LGBQTI on websites to aid in recruitment efforts. Future studies may use other aspects of training experiences within the residency program (such as training opportunities within subspecialities) as a control group and compare to our findings in order to account for the possibility that some aspects of our findings may be due to marketing limitations and strategies of residency program websites. More importantly, future studies could focus on determining the importance of LGBQTI training to residency applicants, how residency website content correlates with training experiences, and on describing the role of social media platforms in the residency recruitment process.

### Electronic supplementary material

Below is the link to the electronic supplementary material.

Supplementary Material 1

## Data Availability

The datasets used and/or analyzed during the current study are available from the corresponding author on reasonable request.

## Abbreviations

LGBTQI: Lesbian, gay, bisexual, transgender, queer, intersex
OB-GYN: Obstetrics and gynecology
DEI: Diversity, equity, and inclusion
