# Supplementary material for: LGBTQI content on obstetrics and gynecology residency websites
Source: BMC Med Educ. 2023 Nov 12;23:854. doi: 10.1186/s12909-023-04624-3 (PMC10642032; doi:10.1186/s12909-023-04624-3)
Supplement: Supplementary file 1 — Supplementary Material 1 [file 12909_2023_4624_MOESM1_ESM.docx]

**Table 1 (Supplementary). Association between mention of LGTBQI didactics and rotations on US OB-GYN residency websites and program characteristics**

|  | | **Mention of LGBTQI rotations and didactics**  **(n=23)** | **No mention of LGBTQI rotation and didactics**  **n (=264)** | p value* |
| --- | --- | --- | --- | --- |
|  | | n(%) | n(%) |  |
| **Region** | |  |  | 0.009 |
| Northeast | | 8 (9.9) | 73 (90.1) |  |
| Midwest | | 2 (2.8) | 69 (97.2) |  |
| South | | 5 (5.2) | 92 (94.9) |  |
| West | | 8 (21.6) | 29 (73.4) |  |
| **State Party** | |  |  | 0.255 |
| Democratic | | 14 (10.9) | 114 (89.1) |  |
| Republican | | 5 (6.4) | 73 (93.6) |  |
| Swing | | 4 (5.0) | 76 (95.0) |  |
| **Program Type** |  | |  | 0.148 |
| Community | | 2 (3.9) | 50 (96.1) |  |
| University | | 15 (12.1) | 109 (87.9) |  |
| CB University | | 6 (5.8) | 98 (94.2) |  |
| Military | | 0 (0.0) | 7 (100.0) |  |
| **Religious** | |  |  | 0.673 |
| No | | 22 (8.5) | 238 (91.5) |  |
| Yes | | 1 (4.0) | 24 (96.0) |  |
| **Website Extent** | |  |  | 0.033 |
| Simple | | 0 (0.0) | 24 (100.0) |  |
| Moderate | | 1 (1.8) | 54 (98.2) |  |
| Complex | | 22 (10.6) | 186 (89.4) |  |
| **# of Residents** | |  |  | 0.021 |
| <16 | | 1 (1.6) | 63 (98.4) |  |
| 16-24 | | 12 (7.8) | 143 (92.3) |  |
| >24 | | 10 (14.7) | 58 (85.3) |  |
| **# of Fellowships** | |  |  | <0.001 |
| 0 | | 2 (1.3) | 156 (98.7) |  |
| 1 or 2 | | 9 (13.4) | 58 (86.6) |  |
| 3+ | | 12 (19.4) | 50 (80.6) |  |
| **Sex of Program Director** | |  |  | 0.021 |
| Male | | 4 (4.0) | 96 (96.0) |  |
| Female | | 19 (10.4) | 163 (89.6) |  |
| Unknown | | 0 (0.0) | 5 (100.0) |  |
| **Sex of Chair** | |  |  | 0.004 |
| Male | | 9 (6.3) | 134 (93.7) |  |
| Female | | 13 (15.9) | 69 (84.1) |  |
| Unknown | | 1 (1.6) | 61 (98.4) |  |

LGBTQI: lesbian, gay, bisexual, transgender, queer, intersex. Values are n (%). Percentages are row percentages. * P-values based on Chi-square
